# Spatio-temporal patterns of ovarian development and *VgR* gene silencing reduced fecundity in parthenogenetic *Artemia*

**DOI:** 10.1098/rsob.230172

**Published:** 2023-11-15

**Authors:** Hu Duan, Xuanxuan Shao, Wei Liu, Jianhai Xiang, Namin Pan, Xuehui Wang, Guoru Du, Ying Li, Jiaping Zhou, Liying Sui

**Affiliations:** ^1^ Asian Regional Artemia Reference Center, College of Marine and Environmental Sciences, Tianjin University of Science and Technology, No. 29, 13th Avenue, Tianjin 300457, People's Republic of China; ^2^ Key Laboratory of Marine Resource Chemistry and Food Technology, Ministry of Education, Tianjin University of Science and Technology, No. 29, 13th Avenue, Tianjin 300457, People's Republic of China; ^3^ Research Center of Modern Analytical Technology, Tianjin University of Science and Technology, No. 29, 13th Avenue, Tianjin 300457, People's Republic of China; ^4^ Key Laboratory of Experimental Marine Biology, Institute of Oceanology, Chinese Academy of Sciences, 7 Nanhai Road, Qingdao 266071, People's Republic of China; ^5^ Tianjin Fisheries Research Institute, Tianjin 300221, People's Republic of China

**Keywords:** ovarian development, oogenesis, germline stem cell, parthenogenetic *Artemia*, RNA-seq, *Ap-VgR* function

## Abstract

The halophilic zooplankton brine shrimp *Artemia* has been used as an experimental animal in multidisciplinary studies. However, the reproductive patterns and its regulatory mechanisms in *Artemia* remain unclear. In this study, the ovarian development process of parthenogenetic *Artemia* (*A. parthenogenetica*) was divided into five stages, and oogenesis or egg formation was identified in six phases. The oogenesis mode was assumed to be polytrophic. We also traced the dynamic translocation of candidate germline stem cells (cGSCs) using EdU labelling and elucidated several key cytological events in oogenesis through haematoxylin and eosin staining and fluorescence imaging. Distinguished from the ovary structure of insects and crustaceans, *Artemia* germarium originated from ovariole buds and are located at the base of the ovarioles. RNA-seq based on five stages of ovarian development identified 2657 upregulated genes related to reproduction by pair-to-pair comparison. *Gbb*, *Dpp*, *piwi*, *vasa*, *nanos*, *VgA* and *VgR* genes associated with cGSCs recognition and reproductive development were screened and verified using qPCR. Silencing of the *VgR* gene in *A. parthenogenetica* (*Ap-VgR*) at ovarian development Stage II led to a low level of gene expression (less than 10%) within 5 days, which resulted in variations in oogenesis-related gene expression and significantly inhibited vitellogenesis, impeded oocyte maturation, and eventually decreased the number of offspring. In conclusion, we have illustrated the patterns of ovarian development, outlined the key spatio-temporal features of oogenesis and identified the negative impacts of VgR gene knockdown on oogenesis using *A. parthenogenetica* as an experimental animal. The findings of this study also lay a foundation for the further study of reproductive biology of invertebrates.

## Graphical abstract

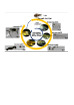

## 1. Introduction

Reproductive development is vital for the development and propagation of multicellular organisms [1]. Sequential events during ovarian development and oogenesis, also known as egg formation, are critical for female reproduction [2]. Egg formation in oviparous females have been well studied, especially in classical ‘model’ organisms and some insects [3,4]. However, the subject of evolutionary developmental biology (evo-devo) has become an emerging field that requires a broader picture in the context of high biodiversity [5,6]. Ovarian development in bisexual species generally includes mature follicle generation, oestrogen secretion, egg production, fertilization and other important processes [7] that are accompanied by successive cytological events. In *Drosophila*, oogenesis requires extensive interactions between somatic and germline stem cells, and these developmental events occur in the ovaries [8]. Oogenesis predominantly includes primordial germ cell (PGC) formation and oocyte development, differentiation and maturation. These are controlled and regulated by relevant genes and signalling pathways [9]. As a key subject in developmental and reproductive biology, oogenesis has been extensively studied in mammals, insects and crustaceans [10–12]. However, to date, there has been relatively little research on the patterns of reproductive processes and molecular mechanisms in invertebrates, especially for species living in extreme habitats such as those that are hypersaline or hypoxic. To date, this has resulted in a relatively limited understanding of reproductive evolution.

Hypersaline environments have provided an extraordinary habitat for all domains of life on Earth since the Precambrian [13]. The brine shrimp *Artemia* is affiliated with Crustacea, Branchiopoda and Anostraca, thriving in hypersaline water bodies with salinity up to 20%. *Artemia* has several biological advantages and unique life phenomena, such as a transparent body, asexual and bisexual reproduction modes, oviparity and ovoviviparity breeding strategies, and diapause and de-diapause life cycles [14]. Therefore, *Artemia* has been used as an experimental animal in environmental toxicology, oncology, epigenetics and other research fields [15–17]. Compared to bisexual *Artemia*, parthenogenetic *Artemia* (*A. parthenogenetica*) imposes a special reproduction mode. The female accomplishes a process of sexual reproduction in which the unfertilized egg develops without the contribution of the male gamete [16]. This ensures the production of offspring that develops from unfertilized embryos as well repeatability of the experiments. These offspring are considered ‘full clones’ with the same genotype and are genetically identical to their mother [18,19]. For *Artemia*, the subjects of biological research mainly focuses on diapause and de-diapause mechanism [20], viviparous and ovoviviparous reproduction regulation [21] and physiological response to the toxicological exposure [22,23].

In oviparous animals, vitellogenesis is a prerequisite for oviposition and embryo development [24]. Juvenile hormone (JH) signalling plays a crucial role in insect reproduction [25,26]. But whether the arthropod-specific sesquiterpenoid (JH) is present in crustaceans, and the member of downstream gene and the molecular mechanisms remain elusive. Vitellogenin (Vg) is synthesized in the hepatopancreas of crustaceans [27] and the fat body (FB) of insects [28]. Vg is transported through the blood, binds to vitellogenin receptors (VgRs) located on the plasma membrane of oocytes to form the Vg/VgR complex, and enters the oocyte via endocytosis [29]. *VgR* knockout or mutation can inhibit oocyte maturation in insects or cause sterility in birds, indicating that VgR plays a crucial role in ovarian development in oviparous animals [30,31]. To date, researchers have focused on other genes that play key roles in oogenesis, such as *Dpp* (Decapentaplegic)and *Gbb* (Glass bottom boat) in the BMP signalling pathways, as well as *vasa* (Vasa intronic), *nanos* (Nanos homologue) and *piwi* (P-element-induced wimpy) in reproductive stem cells. Among these, *Dpp* and *Gbb* mainly promote the growth of follicular cells or regulate the synthesis of JH by inhibiting the gene encoding JH acid O-methyltransferase [32,33]. Silencing of *vasa*, a marker of reproductive stem cells, changed the morphology of ovaries and oocytes and reduced the number of eggs in the Japanese blood-sucking worm *Schistosoma japonicum* [34]. *nanos* and *piwi* are important in regulating the expression of germline stem cells [35]. To date, no studies have been conducted on the regulatory genes, pathways, functions and molecular mechanisms of the genes involved in *A. parthenogenetica* oogenesis.

Our study has reported the morphological and tissue characteristics of the ovarian development process, clarified the cytological details of oogenesis. We also screened candidate genes that may be involved in the ovarian development and oogenesis in *Artemia* with a focus on the function of the *Ap-VgR* gene in the oogenesis process*.* The aim of this study is to provide insights for understanding the mechanism of ovarian development in reproductive biology and to establish a platform for studying the life processes of invertebrates.

## 2. Material and methods

### 2.1. *Artemia* rearing

*Artemia parthenogenetica* cysts from Aibi Lake, China, were obtained from the Asian Regional Artemia Reference Center (ARARC), Tianjin University of Science and Technology, China. Approximately 0.2 g cysts were incubated in a conical tube containing 200 ml dilute brine water with a salinity of 3% under constant hatching conditions at 28°C with 2000 Lux illumination and continuous aeration. After 24 h, *Artemia* nauplii were collected and transferred to a rectangular plastic tank containing 16 l of diluted brine with a salinity of 7%. The initial density was 130 individuals l^−1^. The animals were fed twice daily with *Chlorella* sp. The water was renewed once weekly. The ovary morphology of *Artemia* was observed under a stereoscope (SZ680, China) when the oocyst primordia appeared.

### 2.2. Chemical dyeing

To examine the structure of ovarian development, *Artemia* were fixed in 4% paraformaldehyde (Acros Organics, Lot: A0372619) diluted with PBS (6.6 mM Na_2_HPO_4_/KH_2_PO_4_, 150 mM NaCl, pH 7.4) for 2 h. The ovaries were dissected using ophthalmic forceps (Spi-Swiss) and incubated with 0.5% Triton X-100 (Sigma, Lot: SLBP6453V) for 25 min. After rinsing with PBS, the ovaries were stained with phalloidin (Solarbio, 1029O011) and Hoechst 33342 (Thermo Fisher, Lot:1681305) for 4 h. An anti-fluorescence quenching agent (Beyotime, P0126) was added to prevent fluorescence quenching. The tissue samples were observed under a fluorescence microscope (Nikon Ti-E, Japan).

### 2.3. HE staining

To examine the histological and cytological features in the process of ovarian development, *A. parthenogenetica* individuals representing five developmental stages were collected. They were then fixed immediately in 4% paraformaldehyde at 4°C for 24–48 h [28]. The tissues were dehydrated, permeabilized, immersed in xylene and paraffin wax (1 : 1, v/v) (Bellancom, Lot: DGBF4846V) overnight, and then were dewaxed and stained with haematoxylin and eosin (HE) (BKMAM, Lot : 20220520; 20220428). The tissue samples were then observed under a microscope (Nikon, Japan).

### 2.4. Immunofluorescence antibody labelling

To describe the dynamic migration pathway of germline stem cells during oogenesis, *A. parthenogenetica* ovaries were dissected using forceps and incubated with a mixture of primary antibodies in a cell incubator (Corning) for 2 h. The mixture comprised 2 × EdU working solution (Thermo Fisher, Lot: 2261449), DMEM (Gibco, Lot: 1897090), penicillin, streptomycin and neomycin (Gibco, Lot: 1894168). Tissues were fixed in 4% paraformaldehyde for 2 h. After rinsing with PBS, the tissues were sealed with 3% BSA (Thermo Fisher Scientific, Lot: SF248474) for 20 min. 0.5% Triton 100 was used to increase the permeability of the antibodies to the cell membrane. Secondary antibodies conjugated with Alexa Fluor 488 (Thermo Fisher, Lot: 2261449) were incubated (1 : 500, v/v) with the tissues for 2 h under dark and moist conditions. Finally, the samples were incubated with Hoechst 33342 at 28°C for 30 min at a ratio of 1 : 2000 (v/v). Anti-fluorescent quenching agent was added after cleaning. Images were acquired using a laser confocal microscope (Zeiss 980, Germany).

### 2.5. RNA extraction and illumina sequencing and data analysis

*Artemia* ovaries at five developmental stages were dissected in cold liquid nitrogen. Thirty individuals at the same developmental stage were pooled. RNA extraction was performed using RNAiso Plus reagent (TaKaRa, AKF0727A). Transcriptome library construction and subsequent sequencing analyses were performed by Novogene Technology Co. Ltd., China. Genbank accession number (SAMN36887526) of all used sequences can be found in NCBI.

The original data obtained from high-throughput sequencing were filtered. Functional unigenes were annotated using seven databases, including Gene Ontology (GO; http://www.geneontology.org/) and the Kyoto Encyclopaedia of Genes and Genomes (KEGG; https://www.kegg.jp/). The structural domains of the genes were analysed using SMART. A phylogenetic tree was constructed by the maximum-likelihood method, using MEGA7 with 1000 bootstrap replicates.

### 2.6. RT-qPCR analysis

To explore the spatio-temporal expression patterns of reproductive genes in *Artemia*, total RNA was extracted from the target tissues, namely those of the intestine, cerebral ganglion and ovary, at five developmental stages using RNAiso Plus reagent (TaKaRa, Lot: AKF0727A). cDNAs was synthesized from 1 µg of total RNA (Thermo Fisher Scientific, Lot: A48571). RT-qPCR was performed using TB GreenPremix Ex Taq II (TaKaRa Lot: AL51019A), and β-actin was used as a reference gene. Primers were designed using Primer 5 software (electronic supplementary material, table S1). Each reaction contained three technical replicates and a template-free control.

### 2.7. RNAi and phenotypic analysis

Microinjection needles were prepared using a micropipette puller (Narishige PC-10, Japan) and 1.0 mm capillary tubes (VitalSense Scientific Instruments Co., Ltd. Lot: B100114N). Double-stranded RNA (dsRNAs) of *VgR* and *EGFP* were synthesized using the T7 RiboMAX Express RNAi System (Promega, USA). The primers used for dsRNA synthesis have been summarized in electronic supplementary material, table S2. After anesthetisation on ice, approximately 2000 ng of dsRNA was injected into the ventral side of the second abdominal segment at ovarian development Satge II using a microinjector (Nikon Eclipse Ti, Japan) fitted with an injection needle. The *Artemia* in the control group was treated with the same dose and volume of ds*EGFP*. Ovaries were sampled on the first, third and fifth days after dsRNA injection, and the total RNA was then extracted. The knockdown efficiency and gene variation were determined using qRT-PCR.

For phenotypic assessment, ovaries were collected when more than 80% of *Artemia* had reached the objective developmental stages. Ovary morphologies and cytological features were determined as described in §2.3. Morphological and cytological variations of the ovaries were observed under a fluorescence microscope (Nikon Ti-E, Japan).

### 2.8. Statistical analyses

The data are presented as mean ± standard deviation. Statistical significance was determined using one-way ANOVA followed by Duncan multiple comparisons at *p* < 0.05 and *p* < 0.01, respectively (SPSS26.0). Statistically significant differences between the two groups were inferred using a *t*-test.

## 3. Results

### 3.1. Tissue characteristics in process of ovarian development

**Figure 1.**
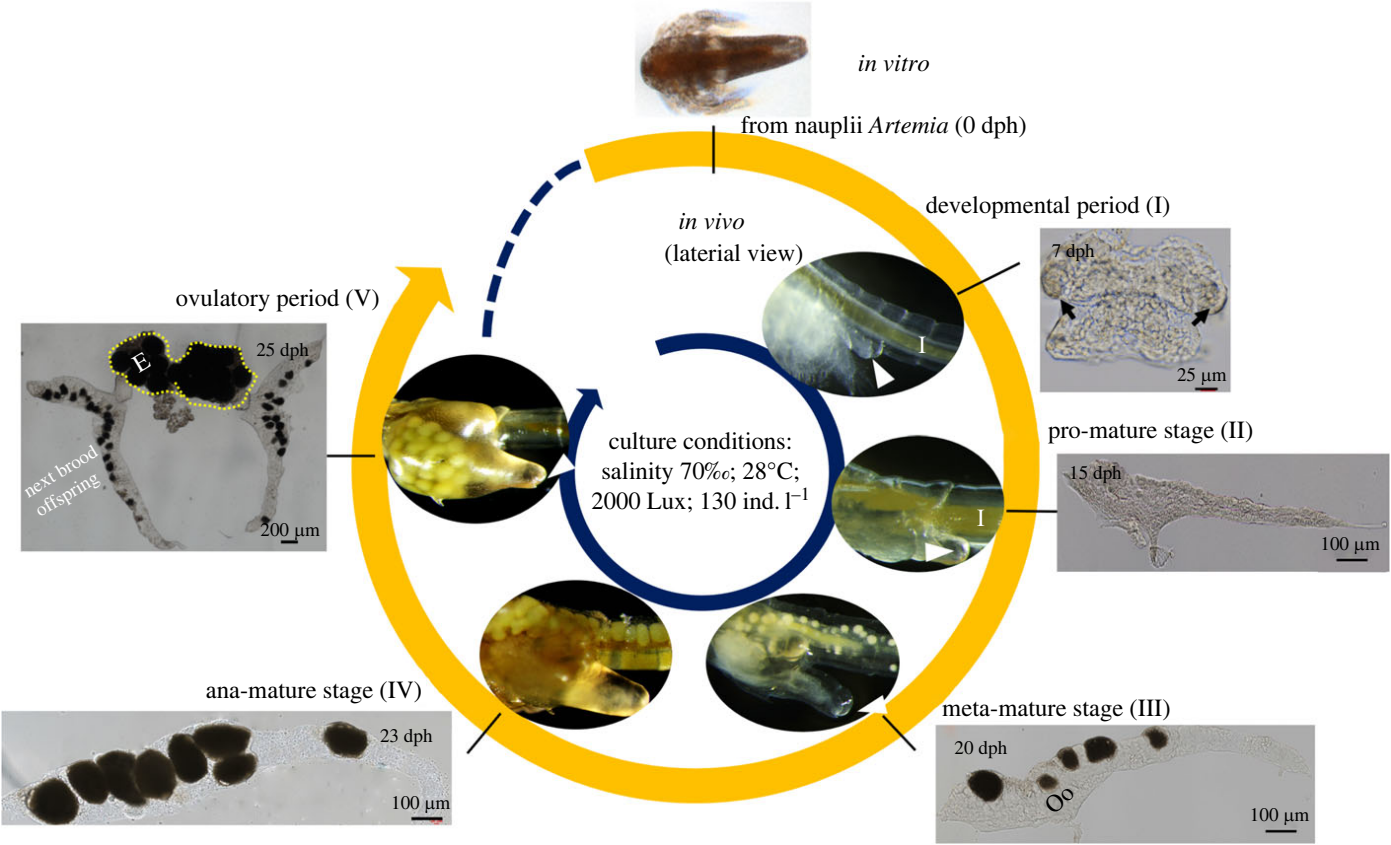
Temporal patterns and morphology of ovarian development in *A. parthenogenetica.* dph, day post-hatching; Ana, anaphasis; E, eggs; I: Intestine; Meta, metaphasis; Oo, oocyte; dark arrows indicate ovariole bud; triangular white arrows indicate ovulatory hole; yellow dotted line indicate oocysts. I, II, III, IV and V indicate different stages of ovarian development, in sequence, as labelled.

After approximately 24 h of hatching, most of the gastrula embryos of *A. parthenogenetica* developed into the Instar I nauplius stage which was considered a starting point for assessing the progress of ovarian development (figure 1, 0 dph, day post-hatching). Within 7–10 dph, approximately 63% of the individuals gradually entered the ovarian developmental period (Stage I), with a prominent pair of ovariole buds (dark arrows, *in vitro*) near the anterior ventral surface of the body. At 15–17 dph, the ovaries reached the mature period, of which approximate 51% individuals were further distinguished into pro-mature stage (II), metaphasis-mature stage (III) and anaphasis-mature stage (IV). The determination of the developmental stage was undertaken according to the ovarian morphology and characteristics such as the ovariole shape, location and features of oocytes (Stages II–IV). At 25–29 dph, the ovaries developed into the ovulatory period (Stage V), and the eggs resided in the oocyst with no longer transparent because of yolk granule accumulation.

### 3.2. Key cytological events of oogenesis

**Figure 2.**
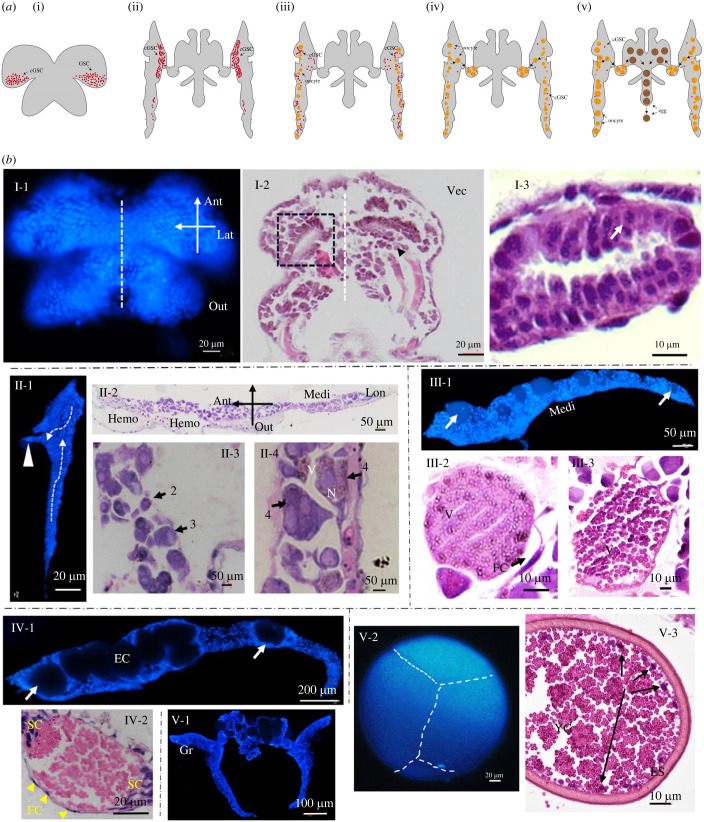
(*a*) Schematic diagram of morphological changes in five stages of ovarian development of *A. parthenogenetica*. Red dot: cGSCs; Yellow circle: oocytes; Brown circle: eggs. (*b*) Cytological characteristics of oocytes accompanied by the ovarian development of *A. parthenogenetica.* Roman numerals I–V represent the five stages of ovarian development. Stage I: dark arrows indicate oogonia in the ovariole. Vec: vector section. Stage II: The white square dots in panel II-1 indicate, migration route of egg formation; Ant, anterior; Hemo, haemocoel; Lon, longitudinal section; Medi, medial surface; N, nucleus; Or, ovarioles; Out, outside. Stage III: FC, follicle cells (dark arrows); V, vitellogenin. Stage IV: SC, support cells; EC, egg chamber; Gr, germarium. White arrows indicate oocytes from different regions of the ovariole. Stage V: ES: eggshell; YG: yolk granules; white arrowhead in panel II-1: oviduct channel.

**Figure 3.**
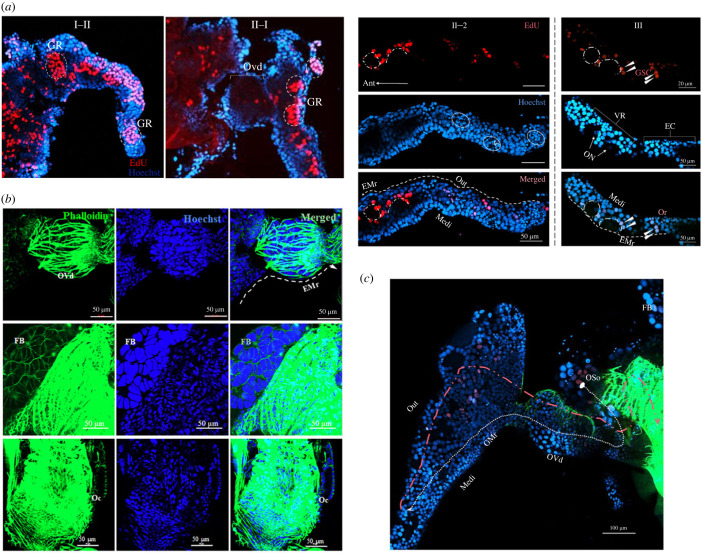
Spatial dynamics of germline stem cells during ovarian development. (*a*) cGSCs translocation. White dotted line indicates direction; EC, egg chamber; EMr, egg formation and migration route; Gr, germarium; Vr, vitellarium; cGSC, candidate germline stem cell; white arrows, cGSCs; ON, oocyte nucleus; OP, oocyst primordium; SC, support cells; OI, oocyte I; Or, ovarioles. (*b*) Structural features of ovaries. FB, fat body; Ovd, oviduct; Oc, oocyst. (*c*) Migration pathways in stem cells. GMr, germline stem cell migration route; white dotted line indicates direction; Medi, medial; Out, outwards; Oso, original site of oogonia.

Based on the observation of the five stages of ovarian development of *A. parthenogenetica*, the morphological diagrams were drawn (figure 2*a*). On this basis, the whole process of egg formation could be further understood. The cytological characteristics of the ovaries were shown by Hoechst and HE staining. The ovaries showed general bilateral symmetry throughout the ovarian developmental process (figure 2*b*, I-1,2, white dotted line). Based on the features of vitellogenesis and the accumulation and distribution of yolk granules observed under optical and fluorescence microscopy, six phases of oogenesis from oogonia to multicellular eggs were distinguished during egg formation (figure 2*b*, I-3, II-3,4, III-2,3, IV-2 and V-2,3). The oocyte was surrounded by follicular cells (figure 2*b*, IV-2, yellow arrows) and support cells (figure 2*b*, IV-2, SC) along the axis of the ovariole, showing that oogenesis of *Artemia* followed a polytrophic oocyte mode.

At Stage I, the ovary was not fully developed, which was transparent with a small volume and lacked paired intact ovarioles (figure 2*b*, I). The first phase oocytes (oogonia) had a high nucleo-cytoplasmic ratio, nearly round shape and highly homogeneous by HE staining (figure 2*b*, I-2,3, dark arrow). The second phase oocytes (figure 2*b*, II-3, dark arrow) occurred at Stage II with an irregular elliptical shape and larger size. The third phase oocytes (figure 2*b*, II-3, dark arrow) were round or polygonal with prominent nucleoli, and the fourth phase oocytes (figure 2*b*, II-4, dark arrow) had an irregular shape with increased cell volume. The oocyte maturation process was characterized by gradually increasing oocyte size and yolk granules, but a decreased nucleo-cytoplasmic ratio (figure 2*b*, III–V).

At Stage III, the fifth phase oocytes were observed. As the ovaries developed, the number of opaque eggs increased. The egg chamber (EC) gradually formed in the ovarioles and contained the eggs that about to maturity. Follicular cells (figure 2*b*, III-2, FC, yellow arrow) were observed in Stage IV, with a row of single follicular cells (figure 2*b*, IV-2) surrounding the oocytes (figure 2*b*). At Stage V, the sixth phase oocytes were fully mature and entered the oocysts, which were enlarged black or grey cells. Before being released by the female, cytoplasmic cleavage occurs to form the multicellular egg (syncytium), which then develops into a gastrula embryo.

### 3.3. Spatial dynamic changes of candidate germline stem cells

As shown by the immunofluorescence images, the ovary of *Artemia* included four parts (figures 1 and 3): a pair of ovarioles (Or), an oviduct (Ovd), an oocyst (Oc) and an ovulatory hole (Oh). The FB was tightly embedded in the ovaries (figure 3*b*,*c*). The candidate germline stem cells (cGSCs) were identified using EdU labelling (figure 3*a*) during Stages I–III of ovarian development. Oocytes in other phases were confirmed based on these results (figure 2*b*) and their spatial position during ovarian development (figure 3). In Stages I and II of ovarian development, the cGSCs of *Artemia* were generated in the germarium (Gr), which contained dividing PGCs and oogonia produced from them. By contrast to the generation, maturation and migration routes of *Drosophila* GSCs, the Gr of *Artemia* was located at the proximal end of the oviduct (figure 3*a*, white dotted circles). The vitellarium (Vr) of *Artemia* was located at the distal end of the oviduct (figure 3*c*). As a pair of ovarioles fully developed, the oocytes increased in number and were surrounded by follicular cells that moved downward and migrated to the Vr, where they continued to develop and mature (figure 3*a*). Meanwhile, the Vr expanded to form a series of ECs in which oocytes were gradually deposited into the yolk proteins. To complete the reproduction, the eggs (gastrula embryos) either developed into nauplii via ovoviviparous reproduction, or were surrounded by a thick shell (ES, figure 2*b*, V-3) and entered a state of diapause (cysts, oviparous reproduction), which were released through the ovulatory hole (figure 1, triangular white arrow).

### 3.4. Transcriptome analysis of ovaries at five developmental stages

In total, 131 442 transcripts and 60 801 unigenes were obtained from the ovary tissues at the five developmental stages (electronic supplementary material, table S3). All the sequences were spliced using BLAST and compared with seven databases, including Pfam, Nr and Swissport, to obtain the corresponding annotation information. Annotations from databases are shown in electronic supplementary material, table S4 and figures S1–S5.

**Figure 4.**
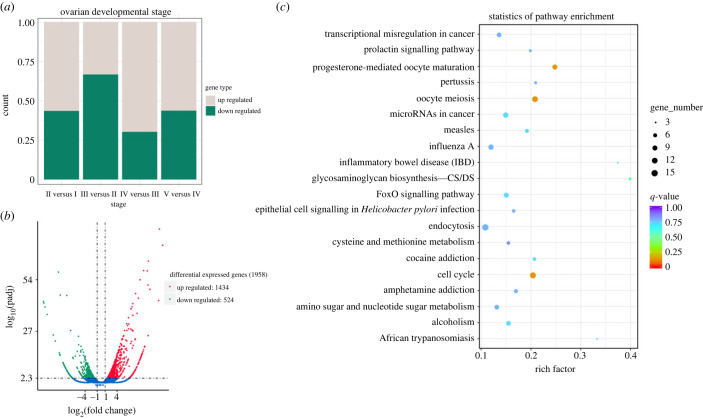
(*a*) Differential expressed genes during the ovarian development of *Artemia*. (*b*) Volcanic plot and (*c*) scatter plot of KEGG enrichment of DEGs in the ovaries of *Artemia* at developmental Stage IV versus Stage II.

To clarify the gene expression profiles and molecular functions of key genes during ovary development process, 28 459 differential expressed genes (DEGs) were identified using transcriptome analysis at five ovarian developmental stages (figure 4*a*). On this basis, combined with the morphological changes in the five stages of ovarian development,

We conduct a statistical analysis of DEGs in Stage IV versus Stage II of ovarian development. DEGs obtained in Stage IV versus Stage II ovarian development were statistically analysed. The volcano map shows the number of common and unique DEGs between the two groups (figure 4*b*). KEGG pathway analysis showed that 121 pathways were enriched in Stage IV compared to Stage II, and each pathway represented one or more biological processes. These pathways are relevant to progesterone-mediated oocyte maturation, the cell cycle and oocyte meiosis which can provide important information for subsequent studies on reproductive development (figure 4*c*). The results of DEGs analysis for other ovarian developmental stages are shown in electronic supplementary material, figures S4 and S5.

### 3.5. Expression profile of transcripts involved in reproduction-related genes

**Figure 5.**
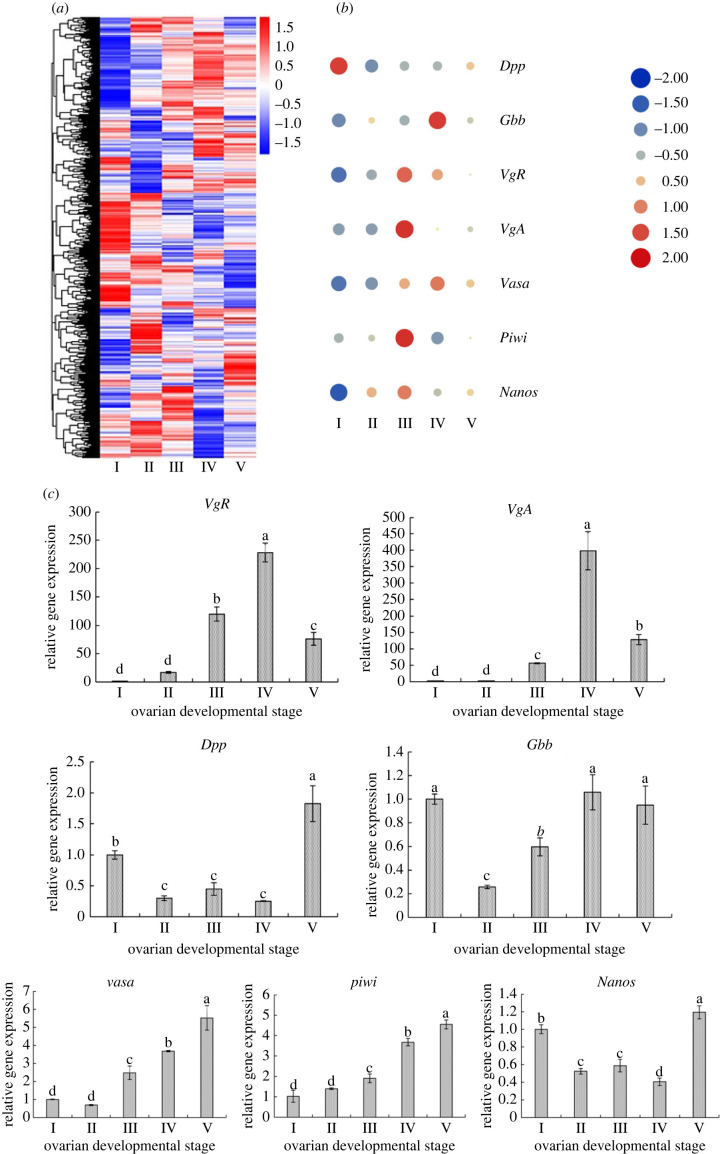
Analysis and qPCR verification of DEGs. (*a*) Clustering heat map of DEGs in ovaries at five developmental stages of *Artemia*. (*b*) Differential expression of the selected reproductive genes. (*c*) Variation in gene expression levels at five ovarian developmental stages of *Artemia*. Data are expressed as the mean ± s.d. (*n* = 30). Different alphabets on the bars represent the significant difference among the groups (*p* < 0.05 or *p* < 0.01).

The FPKM value of each comparison group in the differential gene set of all comparison groups was calculated and these were used for hierarchical clustering analysis of the DEGs expression level (figure 5*a*). Some reproductive DEGs were screened, and the key genes in BMP signalling pathway (*Dpp* and *Gbb)* and JH signalling pathway (*VgR* and *VgA)* and other reproduction-related genes (*vasa*, *piwi* and *nanos*) were further verified by qRT-PCR (figure 5*b*). This showed that the expression profiles of these genes were consistent with transcriptome sequencing results (figure 5*c*). At all sampling times, transcript expression levels varied among the seven genes. However, a higher gene expression level of *VgR*, *VgA*, *Dpp* and *Gbb* were obtained, peaking at Stage III and/or Stage IV (*p* < 0.05 or *p* < 0.01). The expression profile of these genes provided clues and a basis for implementing *Artemia* RNAi manipulation when appropriate.

### 3.6. Structure and phylogenetic analysis of *VgR* in *A. parthenogenetica* (*Ap-VgR*)

**Figure 6.**
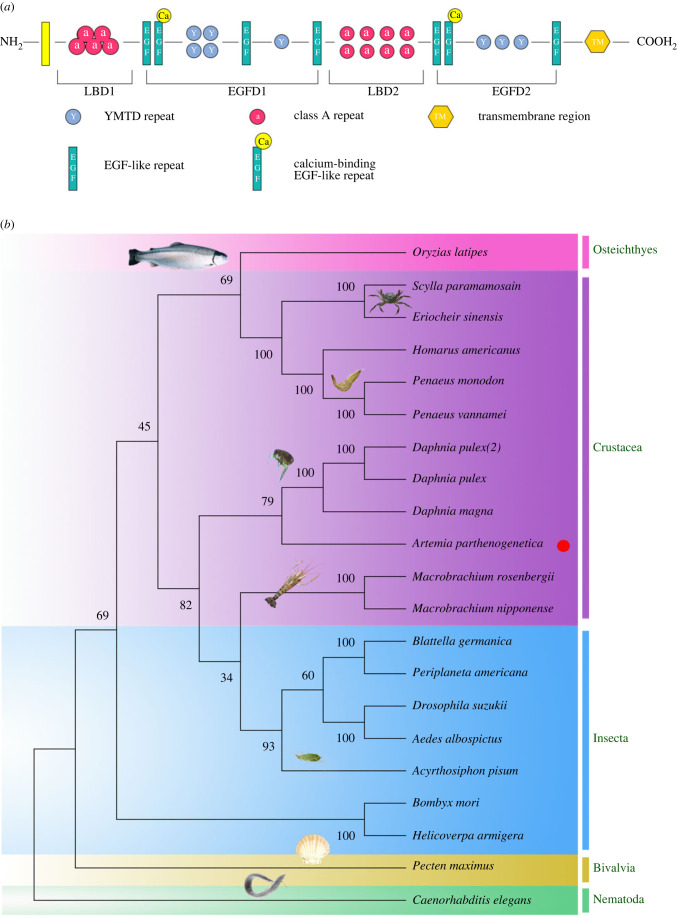
Protein structure of VgR in *Artemia* and phylogenetic analysis of *VgR*s in related species. (*a*) The structure domains of *Ap-VgR* protein of *Artemia*, including ligand-binding domain (LBD), EGF-precursor homology domain (EGFD) and transmembrane domain (TM). (*b*) Phylogenetic tree of *VgR*s in various oviparous species.

In line with the *VgR*s of other crustaceans [36], *Ap-VgR* contained three representative conserved modular elements of the low-density lipoprotein receptor (LDLR) superfamily (figure 6*a*). The ligand-binding domain (LBD) mediates receptor binding to ligands and contains Class A repeats (LDLa). The EGF-precursor homology domain (EGFD) mediates dissociation of receptor and liganded, and consists of YWXD motifs and epidermal growth factor (EGF) repeats. At the carboxyl terminus, transmembrane domain (TM) formed a transmembrane α-helix, which anchored the receptor to the plasma membrane. However, *Ap-VgR* does not contain an O-linked sugar domain (OLSD). These results suggest that *Ap-VgR* may regulate Vg transition into oocytes in a pattern similar to insect *VgR*.

The GenBank accession numbers of the proteins are shown in electronic supplementary material, table S5. Phylogenetic analysis showed that *VgR*s from various oviparous species were grouped into four clades affiliated with *vertebrates*, *arthropods*, *mollusc* and *nematodes*, and *Ap-VgR* was fitted into a clade of crustaceans and was more closely related to Daphniidae (figure 6*b*).

### 3.7. Expression profile of corresponding genes after *Ap-VgR* slicing

**Figure 7.**
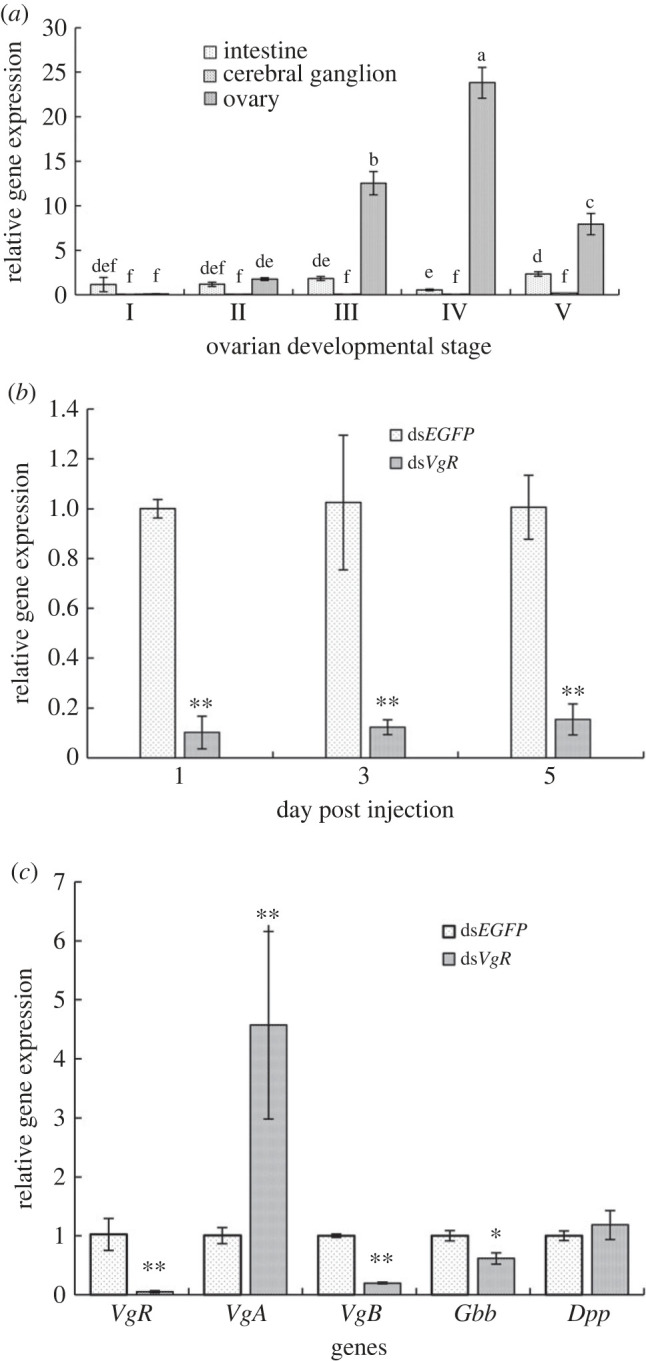
Profiles of *VgR* expression in the process of ovarian development of *Artemia*. (*a*) Expression levels of *Ap-VgR* mRNA in intestine, cerebral ganglion and ovary of at five ovarian development stages (I–V). (*b*) Expression level of *Ap-VgR* mRNA on the first, third and fifth day post injection of ds*EGFP* and ds*VgR*. (*c*) Expression levels of *VgA*, *VgB*, *Gbb* and *Dpp* mRNA on the first day post dsRNA injection. Data are expressed as mean ± s.d. (*n* = 30). Different letters represent significant difference among the groups (*p* < 0.01). Asterisks indicate significant differences between two groups (*p < 0.05, **p < 0.01).

*Ap-VgR* transcript expression was detected in the intestine, cerebral ganglion and ovary of *Artemia* using qPCR (figure 7*a*). The results showed that *Ap-VgR* was specifically expressed in the ovary but was hardly detected in the other two tissues. The expression level of *Ap-VgR* increased continuously and reached the highest at ovarian developmental Stage IV. These results were consistent with the trends observed in the RNA-seq data.

In this study, RNAi assays were conducted to determine the functions of *Ap-VgR* in the oogenesis of *Artemia*, and dsRNA was injected at the pro-mature stage according to the transcriptome results (figure 4). Compared to the ds*EGFP* group (control), the level of *Ap-VgR* in the ds*VgR*-treated group decreased by 89.8%, 90.2% and 85.2% on the first, third, fifth day post injection, respectively (figure 7*b*). The effect of *Ap-VgR* RNAi on the expression of oogenesis-related genes *VgA*, *VgB*, *Dpp* and *Gbb* was studied further. Compared to the ds*EGFP* group, the expression of *VgA* was significantly increased by silencing *Ap-VgR*. The expressions of *VgB* and *Gbb* were downregulated, whereas that of *Dpp* was not significantly altered (figure 7*c*).

### 3.8. Phenotypes modulation of oogenesis after *Ap-VgR* knockdown

**Figure 8.**
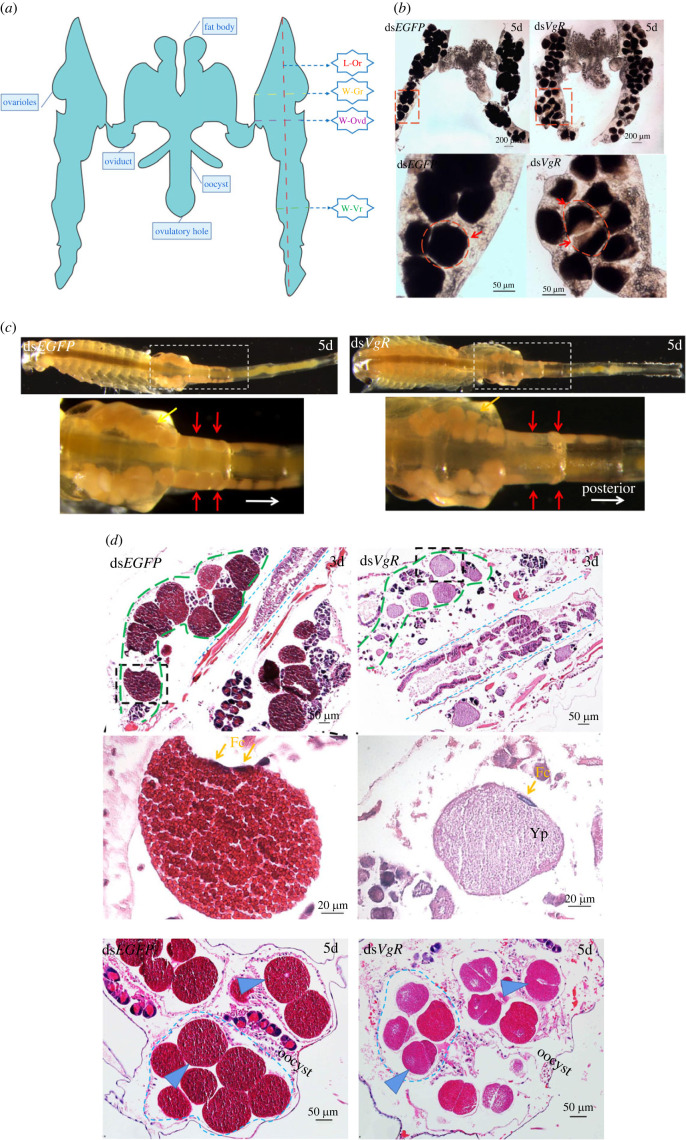
Anatomical and morphological features of *Artemia* ovaries after *Ap-VgR* silencing. (*a*) Schematic figure of mature *Artemia* ovary*.* L-Or, length of the ovarioles; W-Gr, width of the germarium; W-Ovd, width of the oviduct; W-Vr, width of the vitellarium. (*b*) *In vitro* observation of *Artemia* ovary in ds*VgR* and ds*EGFP* group. Red arrows indicate the eggs. (*c*) *In vivo* observation of *Artemia* ovary in ds*VgR* and ds*EGFP* group. The white arrows dots show the migration route of egg formation. Red arrows indicate the eggs distributing symmetrically in ovarioles. (*d*) Cross section of HE-stained ovaries. Green arrows indicate the eggs in the ovariole. Yellow arrows indicate the follicular cells.

The ovaries of *Ap-VgR* knockdown individuals exhibited clear phenotypic changes (figure 8*a*). *In vivo* observations showed that egg development in the ovary was delayed by the third day after ds*VgR* injection. On the fifth day after the ds*VgR* injection, abnormal egg development, disordered distribution of eggs in the ovarioles and a reduced number of eggs were observed. *In vitro* observations showed that the ovaries of the ds*VgR* group had a substantial number of deformities and were less opaque (figure 8*b*, lower-right figure). Biometric measurement of the ovarioles showed that *Artemia* in the ds*VgR* group had significantly shorter ovarioles and shorter widths of the germarium, oviduct and vitellarium (*p* < 0.05). This further demonstrated that the knockdown of *Ap-VgR* delayed ovarian development and ultimately led to reduced fecundity (figure 8*b*,*c* and table 1).

**Table 1.** Biometric measurement of *Artemia* ovarioles after RNAi (*n* = 10). Asterisk indicates a significant difference between two groups (*p* < 0.05).

groups	length of the ovarioles (μm)	width of the germarium (μm)	width of the oviduct (μm)	width of the vitellarium (μm)
ds*EGFP*	1484.46 ± 226.71	232.93 ± 70.39	233.63 ± 44.35	193.89 ± 92.64
ds*VgR*	1384.07 ± 223.71	214.94 ± 119.24	149.19 ± 45.73*	134.42 ± 75.34

**Figure 9.**
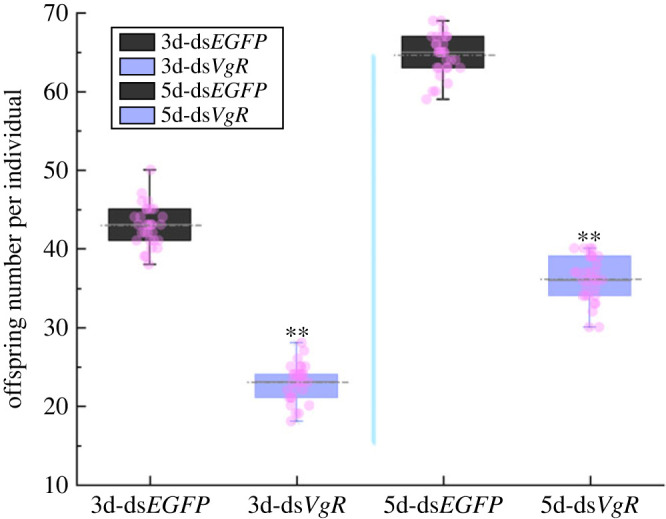
Offspring number per individual of *Artemia* on the third and fifth days after the ovulation period (*n* = 30). Double asterisk indicates a significant difference between two groups (*p* < 0.01).

On the third day post dsRNA injection, HE staining of the ovary showed no pronounced morphological changes in the follicular cells after *Ap-VgR* gene silencing, and all of them had formed unicellular eggs (figure 8*d*). Meanwhile, the yolk protein further accumulated and aggregated in the eggs to form a large number of yolk granules in the ds*EGFP* group (figure 8*d*). However, the eggs in the ds*VgR* group were deformed with less accumulation of yolk granules but were rich in yolk protein. On the fifth day after dsRNA injection, the accumulation of yolk granules in the ds*EGFP* group was complete and typical granular eggs were formed in the oocyst. When the *VgR* gene was knocked down, cleavage was achieved in advance with pronounced unevenness and a substantial number of vacuoles, indicating inferior egg quality. The number of eggs in the ds*VgR* group was significantly lower than that in the ds*EGFP* group (*p* < 0.01) on both third and fifth day after ovulation period (figure 9 and table 1).

## 4. Discussion

The continuation of life from generation to generation through reproduction and development is the biological basis for the proliferation of life [37]. The biological advantages of *A. parthenogenetica*, such as colonial offspring production, relatively short lifespan and reproductive cycle, make it more suitable for the study of reproductive processes and molecular mechanisms [38,39]. In the present study, the spatio-temporal features of ovarian development and oogenesis in *A. parthenogenetica* were clarified by means of *in vivo* and *in vitro* approaches. The FB tissue was first identified in *Artemia*, which is assumed to play a coincident role in promoting vitellogenesis in the fat bodies of insects and the hepatopancreas of crustaceans [40,41]. The FB and the hepatopancreas provide Vg and promote its binding to VgRs.

We found significant differences in the GSC generative sites and migration pathways between *A. parthenogenetica* and *Drosophila* [4,8]. Upon complete ovariole development, GSCs of *Drosophila* generated from the germarium near the terminal filament [42], whereas the oogonia of *A. parthenogenetica* originate from a pair of ovariole buds. Along with ovarian development, cGSCs population labelled by EdU migrate in the form of a cell mass to the corresponding sites where niches regulate egg formation (figures 1 and 3*a*). The formation of cell mass and the cGSCs niche in *A. parthenogenetica* may facilitate successive brood offspring production of *Artemia*, in favour of population sustainability in extreme environments.

Hormone- and gene-mediated regulation of reproductive development has been well studied in insects [43,44]. However, to date, the mechanism of reproductive development in crustaceans in extreme environments has not been explored. The transcriptome platform is an important and convenient tool for providing data supporting the gene and gene network regulation [45,46]. Our data have shown 28 459 DEGs related to ovarian development and oogenesis in *Artemia*, in which genes relevant to the cell cycle and oocyte meiosis, the signalling pathway of insulin and HIF were significantly expressed. This indicated that these genes may regulate and participate in key cyto-events in ovariole formation, cGSCs generation and migration, vitellogenesis and choriogenesis [47]. In this study, *Ap-VgR* was screened and verified based on the DEGs in the transcriptome. *VgR* has been confirmed as a key receptor for vitellogenesis. This is fundamental for oocyte development in oviparous animals and serves as a key preparatory stage for establishing energy reserves and biomolecules for embryonic development [48,49].

We obtained the full-length cDNA of *VgR* in *A. parthenogenetica* and predicted its corresponding protein structural domains (figure 6*a*). In line with other members of the LDLR superfamily, *Ap-VgR* contains three highly conserved regions (LBD, EGFD and TM). However, similar to some insects, such as fire ants [50] and *Drosophila melanogaster* [51], there is a lack of an O-linked Carbohydrate Domain (OLSD) on the plasma membrane surface in *A. parthenogenetica* VgR. The tissue expression specificity of *VgR* is related to the presence of OLSD structures in VgR [52]. In this study, the expression level of *Ap-VgR* without OLSD was significantly higher in the ovary than in other tissues, whereas the expression level of *VgR* in other species with OLSD was higher in non-ovarian tissues [53,54]. The natural absence of VgR in OLSD can cause familial hypercholesterolaemia in humans [55]. Therefore, the OLSD structure in VgR could be a potential marker of species reproductive specificity.

RNAi of *VgR* suppresses Vg accumulation in the ovaries of the tiger shrimp *P. monodon* [56] and delays ovary maturation in the freshwater shrimp *M. nipponense* [57]. However, the phenotypic details of *VgR* regulation during ovarian development are not well understood and remain controversial in different crustacean species [56,57]. In the present study, the expression of *Ap-VgR* was specifically detected in the ovaries of *Artemia* (figure 7), which is consistent with most insects [58,59]. Our results illustrated that the expression levels of *Ap-VgR* in the ovary increased continuously from ovarian developmental Stage I to Stage IV (figure 5*b*), indicating that the exogenous Vg absorption process is similar to that in other oviparous animals which exhibit exogenous vitellogenesis, such as fish [60] and insects [61]. However, the expression levels of *Ap-VgR* decreased rapidly during the ovulatory period (Stage V), which was in accordance with the increased yolk consumption. These yolk reserves further sustain the survival of non-feeding *Artemia* nauplii for 3–4 d post-hatching.

In the present study, the potential molecular mechanisms underlying the onset of vitellogenesis, and offspring production were demonstrated through *Ap-VgR* regulation in *Artemia*. The abnormal eggs resulting from ds*VgR* injection and relatively low *Ap-VgR* expression over time indicate that *Ap-VgR* knockdown had a sustained inhibitory effect on yolk granule formation and led to a significant decrease in offspring production (figure 9). Multiple copies of the Vg genes were screened from the transcriptome and named using capital letters. Our results indicate *Ap-VgR* knockdown significantly increased *VgA* expression and decreased *VgB* expression. As has been reported in insects, *VgR* silencing may lead to the blocking of the expression of its ligand *VgB* [62]. Meanwhile, the expression of *VgA* gene greatly increased, which seemed to compensate for Vg synthesis to rapidly restore its nutrient reserve [56]. Another explanation is that different *Vg* gene family members are involved in different physiological functions, such as yolk protein formation and play non-nutritional roles [63]. It is necessary to investigate the molecular characteristics of the Vg family as well as their functions in ovarian development, oogenesis, immune response or nutrient delivery. By contrast, *Ap-VgR* silencing reduced the expression of *Gbb*, a key gene in the BMP signalling pathway. BMP plays a regulatory role in the embryonic development of mice [64]; proliferation, differentiation and regeneration of germline stem cells in *Drosophila* [65]; and metamorphosis of insects [33]. We have proposed that the decrease in *Gbb* expression affects development and offspring production by interfering with the normal proliferation and differentiation of germline stem cells.

In conclusion, our study has highlighted the processes and regulatory mechanisms underlying ovarian development and egg formation in *A. parthenogenetica*. The structure and function of *Ap-VgR* gene were more similar to those of insects but different from those of other crustaceans of the same class. It is likely that *Artemia* differentiated into the same class as crustaceans in ancient times, but some physiological characteristics and functions might be more similar to insects. Exploring the reproductive patterns and mechanisms of *A. parthenogenetica* may contribute to understanding the evolutionary route of animal reproduction from the sea to land, and how *Artemia* adapt to environmental changes and maintain population stability. With the publication of the genome of *Artemia franciscana* [66,67] and the completion of whole-genome sequencing of *A. parthenogenetica* (data not shown, ARARC sequenced), it is possible to find more accurate and detailed molecular evidence to interpret the role of *Artemia* in the evolution of species at the omics level.

## Data Availability

Supplementary material is available online [68].
